# Evaluation of the Quality of Life in Adult Cancer Survivors (QLACS) scale for long-term cancer survivors in a sample of breast cancer survivors

**DOI:** 10.1186/1477-7525-4-92

**Published:** 2006-12-01

**Authors:** Nancy E Avis, Edward Ip, Kristie Long Foley

**Affiliations:** 1 Department of Social Sciences and Health Policy, Division of Public Health Sciences, Wake Forest University School of Medicine, Winston-Salem, North Carolina, USA; 2 Department of Biostatistical Sciences, Division of Public Health Sciences, Wake Forest University School of Medicine, Winston-Salem, North Carolina, USA

## Abstract

**Background:**

This paper evaluates psychometric properties of a recently developed measure focusing on the health-related quality of life (HRQL) of long-term cancer survivors, the Quality of Life in Adult Survivors scale (QLACS), in a sample of breast cancer survivors. This represents an important area of study, given the large number of breast cancer patients surviving many years post diagnosis.

**Methods:**

Analyses are based on an 8-year follow-up of a sample of breast cancer survivors who participated in an earlier study conducted in 1995. Participants were re-contacted in 2003 and those who were reachable and agreed to participate (n = 94) were surveyed using a variety of measures including the QLACS. Additional follow-up surveys were conducted 2 weeks and one year later. Psychometric tests of the QLACS included test-retest reliability, concurrent and retrospective validity, and responsiveness.

**Results:**

The QLACS domain and summary scores showed good test-retest reliability (all test-retest correlations were above .7) and high internal consistency. The Generic Summary Score showed convergent validity with other measures designed to assess generic HRQL. The Cancer-Specific Summary score exhibited divergent validity with generic HRQL measures, but not a cancer-related specific measure. The QLACS Cancer-Specific Summary Score demonstrated satisfactory predictive validity for factors that were previously shown to be correlated with HRQL. The QLACS generally demonstrated a high level of responsiveness to life changes.

**Conclusion:**

The QLACS may serve as a useful measure for assessing HRQL among long-term breast cancer survivors that are not otherwise captured by generic measures or those specifically designed for newly diagnosed patients.

## Background

The importance of quality of life issues for cancer patients is well-recognized by both researchers and clinicians [1-3]. Over the past several decades numerous studies have addressed the physical, emotional, social, and sexual well-being of cancer patients with the focus largely on the period of treatment following diagnosis. With improved early detection and treatment, large numbers of breast cancer patients are now surviving many years post diagnosis. Sixty-four percent of adults diagnosed with cancer today will be alive five years after their diagnosis [4], while 88% of women diagnosed with breast cancer will be alive 5 years after diagnosis and 80% will be alive after 10 years [4]. The large number of women surviving many years post breast cancer diagnosis has heightened interest in studying long-term effects of cancer on quality of life [5,6]. Research suggesting that cancer treatments can have long-term physical, psychological, sexual and cognitive effects that may influence quality of life has added to this interest [5,7-12].

Long-term consequences of breast cancer include issues present after diagnosis and treatment that linger, but also new concerns that develop over time [9]. Conditions that continue after treatment are pain and fatigue [10,11,13,14], sexual problems [8] and appearance and body-image concerns [6]. Psychological dysfunction can also be a problem [5,8,15]. Newer issues that may develop include insurance concerns, worry about the health of children, and worry about the family's future in the event of recurrence [6,11,15]. Late physical effects of cancer treatment, such as cardiac toxicity or development of second malignancies have also been identified [16,17]. It is also important to recognize that it is not uncommon for people to report positive outcomes of cancer such as better personal relationships, a change in priorities, and greater appreciation for life [18-22].

A number of cancer-specific health-related quality of life (HRQL) measures have been developed, such as the Functional Adjustment to Cancer Therapy (FACT) [23], European Organization for Research and Treatment of Cancer (EORTC) [24], Functional Living Index-Cancer (FLIC) [25], and the Cancer Rehabilitation Evaluation System (CaRES) [26]. These measures, however, were designed to capture acute effects of being newly diagnosed with cancer and the immediate effects of surgery and treatment and may not be appropriate for use with long-term survivors.

Despite the importance of HRQL for long-term survivors, there are currently only two HRQL measures designed specifically for long-term survivors, one of which is a modification of the other [15,27]. Both of these scales have limitations in terms of item wording and HRQL domains. The Quality of Life in Adult Cancer Survivor scale (QLACS) is a recently developed measure specifically focusing on the quality of life of long-term cancer survivors [22] that was developed in response to these limitations. The QLACS is based on a conceptualization of cancer-related quality of life provided by Gotay et al. [2] as the state of well being that is a composite of two components: the ability to perform everyday activities that reflect physical, psychological, and social well-being; and patient satisfaction with levels of functioning and control of the disease. This conceptualization takes into account both functioning and patient satisfaction with functioning and views QOL as a multidimensional construct.

We have previously described the development of the QLACS and some of its psychometric properties (e.g., internal consistency, validity) [22,28]. Initial psychometric evaluation has shown that the instrument has good reliability and face and content validity. The present paper provides an additional evaluation of the QLACS based on data from a follow-up study of breast cancer survivors. This study examined test-retest reliability, responsiveness to change, and additional validity of the QLACS.

## Methods

### Study design and sample selection

The present analyses are based on an 8-yr. follow-up of a sample of younger breast cancer survivors who participated in an earlier study [29,29]. The original study was funded as part of a project to specifically study younger women (≤ age 50) with breast cancer. In the original study, women diagnosed with breast cancer within the past 3 years were recruited from six hospitals in the Greater Boston and New Hampshire areas in 1995. At that time, each institution reviewed their medical records and identified all women who were diagnosed with their first breast cancer in the previous 3 years, were at least 4 months post diagnosis, and were aged 50 or under at the time of diagnosis. Only women who had stage I, II, or III breast cancer were included. The Institutional Review Board of all Institutions reviewed and approved the protocol. The total sample of completed interviews was 202 women. Women were between 25 and 50 years at diagnosis with a mean of 42 years. A description of this initial study and the study sample are reported elsewhere [29].

**Figure 1:**
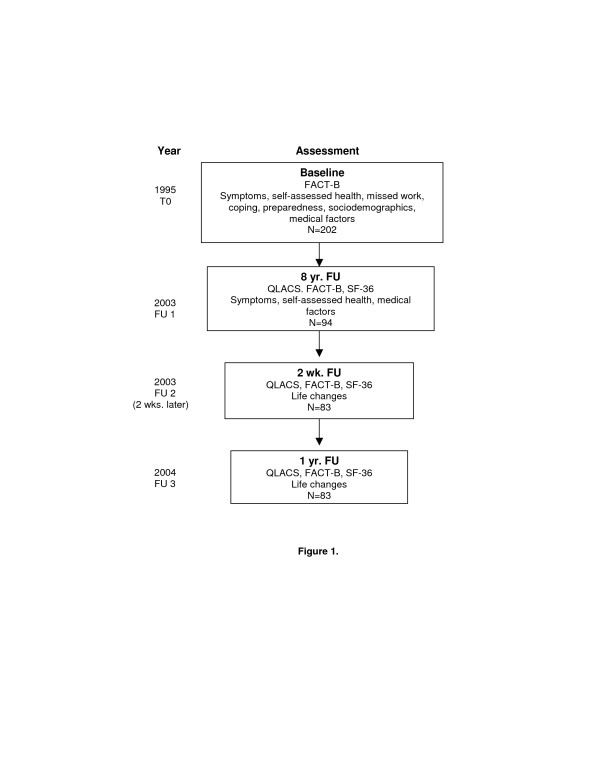
Timeframe for Assessments.

In 2003 these women were recontacted to participate in a follow-up study of long-term cancer survivorship specifically designed to obtain additional psychometric properties of the QLACS. Women were first sent a letter asking if they could be recontacted. Of these women, 29 were discovered to be deceased and 8 refused further contact. The remaining women were then sent a cover letter describing the study, along with the survey instrument and a stamped return envelope. We attempted to locate as many women as possible through use of address correction requests, website searches, and telephone books. After 2 weeks, the survey was re-mailed via Federal Express to those who had not returned their survey. Use of Federal Express provided verification of whether a correct address was available. Participants who did not return their survey after two attempts were contacted by telephone, if a phone number was available. Those who completed the survey were then sent an additional survey two weeks later to assess test-retest reliability of the QLACS. One year later study participants were sent a follow-up survey to assess stability and responsiveness of the QLACS. The study timeline is shown in Figure 1.

### Measures

Table 1 shows when each measure was administered.

**Table 1:** Timeframe for measures

	Baseline (T0) 1995 N = 202	FU1 2003 N = 94	FU2 2003 (2 weeks after FU1) N = 83	FU3 2004 (One year after FU1) N = 83
Global QOL (VAS)	X	X	X	X
FACT-B	X	X	X	X
QLACS		X	X	X
SF-36		X		X
Symptoms	X	X		
Self Assessed Health	X	X		
Days Missed Work or Usual Activity	X			
Coping	X			
Preparedness	X			
Sociodemographics	X	X		
Medical factors	X	X		X
Life change items			X	X

#### Quality of Life in Adult Cancer Survivors (QLACS)

The QLACS is designed to measure HRQL among long-term cancer survivors. It contains 47 items and 12 domains; 7 are considered generic and 5 cancer-specific. Generic domains include those that are not necessarily attributable to cancer – Physical Pain, Negative Feelings, Positive Feelings, Cognitive Problems, Sexual Problems, Social Avoidance, and Fatigue. Cancer-specific domains relate specifically to having had cancer and include Financial Problems resulting from cancer, Distress about Family, Distress about Recurrence, Appearance Concerns, and Benefits of Cancer. Previous factor analyses showed that a 7-factor solution for the generic domains was consistent with the definition of the domains [22]. Accordingly, a Generic summary score is formed by adding its 7 constituent domain scores (reversing the score for Positive Feelings). A separate factor analysis for cancer-specific domains showed that Benefit of Cancer did not load with the other domains. As a result, a Cancer-Specific summary score is created by adding the constituent domains scores except Benefit of Cancer. The score for Benefit of Cancer is reported separately. We continue to follow this framework in this report. The QLACS had not been developed at the time of the baseline assessment and was only administered at the follow-up interviews.

#### SF-36

The Medical Outcomes Short-Form Health Survey (SF-36) is a validated generic instrument for measuring current health status [30]. The survey form contains 36 items that cover 8 dimensions of health: physical functioning, role limitations due to physical problems, bodily pain, general health perception, vitality, social functioning, role limitations due to emotional problems, and mental health. The score for each dimension is between 0 (worst) and 100 (best). The SF-36 was administered at follow-up as a comparison measure to the QLACS.

#### Functional Assessment of Cancer Therapy-Breast (FACT-B)

The Functional Assessment of Cancer Therapy for Breast Cancer [31] was used as a cancer-specific HRQL comparison. The FACT-B consists of the following subscales: physical well-being, functional well-being, emotional well-being, social/family well-being, and breast cancer specific concerns. A total FACT-B score is calculated by summing the subscales. The instrument has a total of 36 items asking respondents to rate how true each statement is for the past 7 days. Response scales range from 0 (not at all) to 4 (very much). The FACT-B has been shown to have high internal consistency, reliability, and has been well-validated [23]. Since we questioned whether the FACT-B was its relevant for long-term survivors, we asked women to indicate on the questionnaire any items that no longer seemed relevant.

#### Global Quality of Life

A 100 mm Visual Analog Scale (VAS) was used to assess global quality of life. The VAS is a validated, single item measure [26,32] that asks respondents to rate their overall quality of life in the past 2 weeks on a scale of 1–100 where 1 represents the lowest possible QOL and 100 the represents the highest QOL.

#### Life Changes

A list of life change items assessed how various aspects of life changed when compared to a specified time point (e.g., 2 wks. ago or one year ago). These items were included at FU2 and FU3 so that test-retest reliability and responsiveness of the QLACS could be determined according to whether or not women reported life changes. The list was developed to tap areas directly related to the dimensions of the QLACS: physical pain, mood, cognitive problems, sexual problems, social functioning, fatigue, financial problems, family-related concerns about cancer, worries about recurrence, feelings about appearance from having cancer, and feelings about positive aspects of having cancer. The list also included a question on overall QOL. Response options ranged from 1 "quite a bit worse" to 5 "quite a bit better

#### Medical History

Medical/treatment variables at baseline included type of initial surgery (lumpectomy, mastectomy, reconstruction), type of treatment (chemotherapy, radiation, hormonal), current treatment, and recurrence. Other questions inquired about age at diagnosis, time since diagnosis, and time since end of treatment. Subsequent surveys asked about surgeries, treatments, and recurrences since baseline.

#### Other Measures

The following measures were cross-sectionally related to the FACT-B at baseline [29] and used in the present study for the retrospective validity analyses.

#### Symptoms

Women were given a list of 14 symptoms derived from the Breast Cancer Prevention Trial Symptom Checklist [33,34] and asked to rate how bothered they had been by each symptom in the past 4 weeks. Responses ranged from 1 = "not at all" to 5 "very much." Symptoms included in the present analyses were hot flashes, nausea, vomiting, diarrhea, difficulty with bladder control when laughing or crying, difficulty with bladder control at other times, vaginal discharge, vaginal dryness, pain with sexual intercourse, swelling of hands and feet, general aches and pains, unhappiness with appearance, weight gain, and weight loss. Symptom scores were summed to provide an overall score.

#### Self-assessed health

This was the widely used single item where respondents rate their health on a 5-point scale from excellent to poor.

#### Days of work/Usual activity missed

Women were asked to indicate the number of days of work or usual activity that they missed in the first 3 months following diagnosis.

#### Coping

Thirty-two items from the Ways of Coping-Cancer Version (WOC-CA) were used to assess coping strategies [35]. This measure and our modification are described in detail elsewhere [36]. Study participants were asked to indicate how often they had used each strategy in the past six months in attempting to cope with the most stressful part of their breast cancer. Previous factor analyses based on the baseline sample revealed seven scales or coping strategies. Four of these scales were related to HRQL at baseline, as measured by the FACT-B: seek and use social support, positive cognitive restructuring, wishful thinking, and made changes.

#### Preparedness

Three items assessed feelings of preparedness for coping with different aspects of breast cancer. Women were asked how prepared they felt for coping with their breast cancer and treatment in terms of the possible impact on their relationships, how they might feel about their appearance after surgery, and the availability of counseling or support groups. These items were developed specifically for the original project to inform future interventions. A factor analysis revealed one dominant factor. We also found high internal consistency among the items (Cronbach's alpha = 0.79) which were summed to provide a single measure.

#### Sociodemographic variables

Included in the survey were questions on current marital/partner status, income, education, age, and employment status. These items were asked at baseline and the first follow-up.

### Analysis

#### Internal consistency

(Cronbach's alpha) for each subscale of the QLACS was assessed at each follow-up (FU1, FU2, and FU3). **Test-retest reliability **of the QLACS was measured by comparing FU1 and FU2 scores, which were administered 2 weeks apart. Only respondents who reported no important life change in the brief period between the two assessments were included in the test-retest analysis. Both the Pearson correlations and intraclass correlation coefficients (ICC) were used to compare the test-retest reliability.

#### Concurrent validity

of the QLACS was assessed by analyzing correlations between the QLACS domain and summary scores with other HRQL measures: SF-36, FACT-B, and VAS. We hypothesized that the QLACS Generic Summary score would be strongly correlated with the other generic QOL measures (e.g., SF-36, VAS), whereas the QLACS Cancer-Specific Summary score would weakly correlate with these measures. The Cancer-Specific summary score was expected to achieve a higher level of correlation with the Breast Cancer Specific Concerns of the FACT-B, which is targeted for breast cancer patients.

#### Retrospective validity

assesses how well a measure retrospectively relates to factors that are hypothesized to be predictive of the measure. In baseline analyses, we found that the following variables were highly related to HRQL as measured by the FACT-B: the number of days of work or usual activity missed in the 3 months following initial diagnosis; four coping strategies – positive cognitive restructuring, seeking and using social support, wishful thinking, and made changes; and feeling prepared for the impact of cancer [29]. We used these baseline variables to predict QLACS scores at FU1.

#### Responsiveness

to change was examined using change in QLACS domain scores 1-yr. apart (from FU1 to FU3). Domain change scores were compared with direct reports of life changes by respondents. The overall QOL item was used for the QLACS summary scores. Study participants were classified into three groups according to their response to the Life Change question – positive change (quite a bit better and somewhat better), no change (about the same), and negative change (quite a bit worse and somewhat worse). An ANOVA test was performed for the change score from each domain to assess whether the differences between the three groups were significant. The p-value, effect sizes and reliable change index (RCI) [37] were used to measure the magnitude of responsiveness. RCI is defined as the difference normalized by the standard error of measurement. The following guidelines were used to characterize effect sizes: <0.2 small changes; 0.2–0.5 moderate change; above 0.5 large change; above 1.0 very large change [38]. An RCI larger than 1.96 is considered reliable change. Because of the possible asymmetry between positive and negative change, effect sizes and SEM were reported separately for each direction.

## Results

### Sample characteristics

Of the 202 women who completed a baseline survey, 92 women completed follow-up surveys at FU1. Surveys were not completed for the following reasons: deceased (n = 29), unable to locate (n = 53), refused (n = 8), did not return survey (n = 17), and too ill (n = 1). An additional 9 women completed surveys at FU1, but not FU2 or FU3. Our response rate was 78% of those who may have received our mailings (we conservatively assume that those surveys not returned by Federal Express were received by participants) and 45.5% of the original sample. This response rate is comparable to other studies that have re-contacted women after this length of time [39,40]. We compared those who completed the survey at FU1 with the full sample at baseline and found that those who were not reached at follow-up were less likely to be partnered at baseline, but they did not differ on any other sociodemographic or medical factors.

Table 2 shows the characteristics of the analytic sample (FU1). The mean age of the sample was 51 years. Most study participants were Caucasian (96%) and the majority were employed (78%). Time since diagnosis ranged from 8.6 to 11.4 years with a mean of 9.9 years. Thirteen women reported a recurrence since initial diagnosis and seven experienced another cancer. Forty-six percent of women had an initial mastectomy and more than half of these women had reconstruction. An additional 10.6% had a mastectomy following baseline and 18% had reconstruction. Seventy-seven percent reported initial chemotherapy and 66% had radiation initially (some women had both). Following baseline, close to 10% of women had chemotherapy and about 7% had radiation.

**Table 2:** Characteristics of FU1 analytic sample n = 94

**Characteristic**	**% (n)**	**Mean (sd)**
**Sociodemographics**		
Age (years)		51.4 (6.2)
38–44	16.0% (15)	
45–50	21.3% (20)	
51–55	28.7% (27)	
56–61	34.0% (32)	
Current partner status:		
No partner	12.8 (12)	
Marriage-like partner	7.4 (7)	
Married	79.8 (75)	
Educational level:		
High school or less	18.1 (17)	
Some college	56.4 (53)	
Post-college	25.5 (24)	
Employed	77.7 (73)	
**Medical Factors**		
Initial mastectomy/reconstruction:		
No mastectomy	54.2 (51)	
Mastectomy, no reconstruction	21.3 (20)	
Mastectomy, reconstruction	24.5 (23)	
Initial chemotherapy:		
No	23.4 (22)	
Yes	76.6 (72)	
Initial radiation therapy:		
No	34.4 (32)	
Yes	65.6 (61)	
Age at diagnosis (years)		41.61 (6.07)
Time since diagnosis (mean)		9.9 years
Currently undergoing treatment		
No	88.3 (83)	
Yes	11.7 (11)	
Treatment since baseline		
Mastectomy since baseline	10.6 (10)	
Reconstruction since baseline	18.1 (17)	
Chemotherapy since baseline	9.6 (9)	
Radiation since baseline	7.4 (7)	
Recurrence since baseline	13.8 (13)	
Other cancer since baseline	7.4 (7)	
General health (5-point scale)		2.29 (0.95)
Total Symptom Score		26.3 (2.2)
Overall quality of life (visual analogue)		70.0 (20.8)

### Characteristics of the QLACS and reliability

Table 3 shows the mean domain scores and possible floor and ceiling effects of the QLACS at FU1. Domains generally showed low floor or ceiling effects with financial problems being the only domain to demonstrate a floor effect where 61% of the sample reported no financial problems. A comparison between these scores and those of the breast cancer sample from the original QLACS paper revealed the only significant difference was for negative feelings. The present sample was significantly higher on negative feelings (11.5 compared with 9.7; p = .01).

**Table 3:** Descriptive summary of distribution of QLACS summary and domain scores, possible floor and ceiling effects at FU1, test-retest correlation, and ICC between FU1 and FU2,

Domain/Subscale	N	Mean	STD	Min	% Floor	Max	% Ceiling	Test-retest Correlation	ICC
Generic									
Negative Feelings	94	11.5	4.2	4	1.1	27	0	0.78	-
Positive Feelings	94	20.8	5.1	7	0	28	2.1	0.82	-
Cognitive Problems	94	10.6	4.3	4	5.3	24	0	0.75	-
Sexual Problems	91	13.7	6.6	4	6.4	28	2.1	0.89	-
Physical Pain	93	8.5	5.0	4	20.1	26	0	0.89	-
Fatigue	94	12.3	5.6	4	4.3	27	0	0.84	-
Social Avoidance	94	7.9	4.6	4	0	26	0	0.86	-
Summary**	93	75.5	26.3	49	0	167	0	0.91	0.95
Cancer-Specific									
Appearance	94	8.2	4.9	4	29.8	24	0	0.83	-
Financial Problems	93	6.2	3.9	4	60.6	25	0	0.82	-
Distress – Recurrence	94	11.7	5.7	4	10.6	28	1.1	0.85	-
Distress – Family	94	10.9	6.7	4	17.0	28	2.1	0.88	-
Summary	94	37.0	15.7	16	2.13	90	0	0.89	0.98
Benefit of Cancer	94	17.6	6.2	4	2.13	28	4.3	0.82	-

N.B. – Missing percentage are low in general. The domain that contains most missing values is Sexual Problem, in which there are 3 missing values (3.2%). Lower scores equal better quality of life.

**The summary score is computed by added the domain scores except for positive feelings. The positive feelings score is subtracted from 32 and then added to the other domain scores.

All test-retest correlations exceed the commonly used standard of 0.7 for testing reproducibility. The ICCs were respectively 0.95 and 0.98 for the two summary scales. The value of Cronbach's alpha at the three time points for domains ranged from 0.72 to 0.95 (data not shown). Most of the alpha values (33 of 36) were well over 0.8, suggesting that domains had a high level of internal consistency [41].

### Concurrent and retrospective validity

Table 4 shows the results of our concurrent validity analyses. All of the generic QLACS subscales correlated highly with the equivalent subscale of the SF-36. The QLACS Generic Summary (GSS) score was highly correlated with the SF-36 PCS and MSC component dimensions (ρ = -0.70, -0.69 respectively) and with the FACT-G (r = -.81). The correlation between the GSS score and three other measures – VAS, total symptom score, and self-assessed health were similar (ρ = -0.72, 0.58, and 0.67, respectively.)

**Table 4:** Correlation coefficients between QLACS scores and other measures

**QLACS scale**	**Measure**	**Correlation**
Subscales		
Physical Pain	SF36 – Bodily pain	-0.82
Negative Feelings	SF-36 mental health	-0.78
Positive Feelings	SF-36 mental health	-0.81
Cognitive Problems	N/A	-
Sexual Problems	N/A	-
Social Avoidance	SF-36 Social functioning	-0.71
Fatigue	SF-36 Vitality	-0.88
QLACS generic summary score (GSS)	SF-36 PCS	-0.70
	SF-36 MCS	-0.69
	FACTG	-0.81
	VAS	-0.72
	Symptoms	0.58
	Self-Assessed Health	0.67
QLACS cancer specific summary score (CSS)	SF-36 PCS	-0.32
	SF-36 MCS	-0.39
	FACT Breast Cancer Specific Subscale	-0.62
	VAS	-0.32
	Symptoms	0.46
	Self-assessed health	0.38

*The N for correlations is from 88 to 94.

As would be expected, the QLACS Cancer-Specific summary (CSS) score had a weaker correlation with the PCS and MCS component scores (ρ = -0.32, -0.39) and the VAS, total symptom score, and self assessed health (ρ = -0.32, 0.46, and 0.38 respectively). The QLACS CSS score showed a greater correlation with the FACT Breast Cancer Specific Concerns subscale (-0.62), which focuses on breast cancer.

These results suggest that the GSS has convergent validity with measures designed to assess generic QOL and the CSS score exhibits divergent validity with generic QOL measures, but not the FACT- B.

We examined retrospective validity by evaluating how well the QLACS at FU1 correlated with factors that were known to be significantly associated with HRQL, measured 8 years earlier at baseline (T0) by the FACT-B. The results reported in Table 5 show that three of the baseline variables (days work/usual activity missed, wishful thinking, and preparedness) were good predictors of the QLACS CSS score and better predictors of the QLACS CSS than of the FACT-B. Positive cognitive restructuring at baseline was a better predictor of the FACT-B and the QLACS GSS.

**Table 5:** Retrospective correlation between measures at baseline (T0) and measures of QOL at FU01. The number in parenthesis is p-value

Baseline Measure	Generic Summary score	Cancer-Specific Summary score	FACT-B
	r (p)	r (p)	r (p)
Days work/usual activity missed following diagnosis	-0.01 (0.92)	0.25 (0.01)	-0.18 (0.09)
Positive cognitive restructuring	-0.24 (0.02)	-0.13 (0.21)	0.21 (0.04)
Wishful thinking	0.14 (0.20)	0.38 (0.0001)	-0.21 (0.05)
Preparedness for cancer	0.10 (0.34)	0.38 (0.0001)	-0.17 (0.11)
Seek social support	-0.02 (0.81)	0.03 (0.80)	-0.02 (0.82)
Made changes	-0.16 (0.13)	-0.15 (0.17)	0.17 (0.10)

*The N for correlations is from 88–94.

### Responsiveness

For each domain and the summary score, responsiveness was gauged by comparing the QLACS change scores at FU1 and one-year later (FU3) with change in health status The mean change scores, effect sizes, and RCIs are reported in Table 6 by direction of self-reported change – worse, or better. Generally, the QLACS demonstrated a high level of responsiveness to life change. All directions of effect size and RCI are consistent with the direction of change in health. Judging by p-values, four domains would be considered not very responsive: Cognitive Problems, Sexual Problems, Family Distress, and Recurrent Distress, though some of the effect sizes could still be considered moderate.

**Table 6:** Responsiveness in terms of p-value, effect size, and reliable change index in either positive or negative direction

		**Group with negative change**			**Group with positive change**		
Domain	Overall F test in ANOVA p	N	Effect Size	Reliable change index	N	Effect Size	Reliable change index
Generic summary	0.02	9	0.83	2.75	29	-0.09	-0.31
Physical Pain	0.001	21	0.63	1.81	9	-0.18	-0.51
Negative Feelings	0.001	12	0.79	1.77	28	-0.29	-0.65
Positive Feelings	<0.001	12	-0.97	-2.23	27	0.36	0.84
Cognitive Problems	0.21	13	0.34	0.69	5	-0.32	-0.66
Sexual Problems	0.24	11	0.13	0.39	9	-0.38	-1.14
Social Avoidance	<0.001	9	1.07	2.98	9	-0.67	-1.85
Fatigue	0.04	15	0.36	1.00	10	-0.13	-0.36
Cancer-Specific summary	0.023	9	0.66	2.10	30	-0.06	-0.20
Financial Problems	0.048	21	0.52	1.11	15	-0.11	-0.23
Family Distress	0.21	13	0.15	0.42	5	-0.43	-1.23
Recurrence Distress	0.40	14	0.30	0.75	7	0.05	0.13
Appearance Concerns	<0.0001	6	1.43	3.46	7	-0.31	-0.74
Benefit of Cancer	0.032	5	-0.26	-0.62	8	0.66	1.55

## Discussion

Despite the importance of HRQL for long-term survivors, there are currently only two HRQL measures designed specifically for long-term survivors, one of which is a modification of the other. Researchers at the City of Hope National Medical Centre were originators in developing a HRQL measure for long-term survivors. They developed the Quality of Life-Cancer Survivors scale (QOL-CS) based on their conceptualization of HRQL as having four dimensions: physical, psychological, social, and spiritual [27]. Although this scale reflects an attempt to recognize HRQL issues relevant to long-term cancer survivors, it has a number of limitations. Items were based on a small number of cancer survivors and validation of the scale was based on survivors ranging from 4 months to 28 years after diagnosis (thus including newly diagnosed patients). Some items have problematic wording, in that they ask about change but fail to indicate its direction (e.g., "has your illness or treatment caused changes in your self-concept?"). The domains often measure multiple constructs at once (e.g., social interaction includes appearance, sexual functioning, and family distress). Further, several items ask about distress at the time of diagnosis and treatment.

Wyatt and colleagues [15] developed the Long-term Quality of Life (LTQL) questionnaire based on the same conceptual model. Based on data from a sample of female cancer survivors, they conducted a factor analysis, an internal consistency analysis, and determined content validity. These results yielded 34 items loading on 4 factors that are slightly different from the QOL-CS: somatic concerns, spiritual/philosophical view of life, fitness, and social support. While the psychometric approach of the LTQL is an advantage,, some of the items themselves are still problematic and the domains often encompass more than one important aspect of HRQL. For example, the somatic concerns domain includes both body-image problems and pain. Type of cancer and its treatment may well vary in terms of their impact on these two concerns. The broad domains do not allow investigators to look at more specific HRQL areas.

The QLACS was developed in response to these limitations. The analyses reported here suggest that the QLACS has good internal consistency reliability, and adequate concurrent and retrospective validity. Responsiveness to change is encouraging, but needs more testing in other samples.

Important findings of this study include the concurrent and retrospective validity of both the Generic Summary score and the Cancer-Specific Summary score in assessing HRQL of long term cancer survivors. We found that the Generic Summary score tracks well with some existing generic measures, while the Cancer-Specific Summary Score showed a much lower correlation with other generic QOL measures. Compared to other instruments, the Cancer-Specific Summary score exhibits relatively high predictive validity as judged by its associations with number of missed days of work/usual activity following diagnosis, the respondent's feelings of preparedness for dealing with cancer, and coping strategies. For example, the number of days missing work has a strong significant correlation with the QLACS CSS, suggesting that women who tend to miss a high number of workdays or usual activity immediately following diagnosis have a lower QOL several years later. Women who reported being well prepared (responded with 4 or 5 on the preparedness scale) showed substantially better quality of life when projected to several years later in the QLACS CSS measure than the FACT-B. These retrospective validity results suggest that the QLACS had better predictive validity than the FACT-B.

Our qualitative data suggests that some of the FACT-B items may not be relevant for long-term survivors. Despite answering all of the items, 52 women indicated that at least one FACT question was no longer relevant to them. This occurred most frequently for questions about pain (N = 30), side effects of treatment (N = 29), nausea (N = 14), and family communication about illness (N = 14). We should point out, however, that we did not ask respondents to indicate when QLACS questions were not relevant.

It is also worth comparing the QLACS scores in this sample with those of the breast cancer sample in the original QLACS paper. The only domain score that was significantly different was negative feelings, with the younger sample reporting greater negative feelings. This finding is consistent with other research showing greater psychological morbidity among younger women with breast cancer [42-44].

The QLACS was generally responsive to self-reported changes. One finding about this population of long-term survivors is the absence of detectable changes in HRQL in several domains – Cognitive Problems, Sexual Problems, Family Distress, and Recurrence Distress (all p > 0.20), even when a change in these areas had been reported. There may be several explanations for this. While it is possible that the QLACS is not sufficiently responsive in these domains, the absence of detectable changes could also be due to this particular sample or the nature of the life change questions. More clinical measures might show greater responsiveness. We also observe that the instrument tends to be more responsive to negative changes. For example, for respondents reporting "Somewhat worse" or "Quite a bit worse" in wanting to socialize (Social Avoidance domain), the effect size was 2.98, compared to an effect size of 1.85 for the group that reported "Somewhat better" or "Quite a bit better" for wanting to socialize. It is important to continue evaluation of the responsiveness of the QLACS in different samples. To our knowledge, however, we are unaware of responsiveness data by domain for other scales such as the FACT.

There are several limitations of this study. The analyses reported here are based on a follow-up of well-educated, young, white female breast cancer survivors. Studies need to be conducted among more diverse samples of survivors of different ethnicities, ages, and cancers to gather more data on the usefulness of the QLACS for other cancer survivors. Another limitation of this work is the number of women lost to follow-up. Finally, our responsiveness data were limited to self-reported life. More research using clinical measures or clinical trial data to assess responsiveness is desirable. Nevertheless, the QLACS may be a a useful measure for assessing HRQL among long term cancer survivors and warrants further evaluation.

## Conclusion

The QLACS appears to be a promising measure to assess HRQL among long-term breast cancer survivors and warrants further evaluation

## Competing interests

The author(s) declare that they have no competing interests.

## Authors' contributions

NA participated in the design of the study, acquisition of the data, and drafted the manuscript. EI conducted the analyses and helped draft the manuscript. KF contributed to the analyses and drafting the manuscript. All authors read and approved the final manuscript.
